# A Dense Fibrillar Collagen Scaffold Differentially Modulates Secretory Function of iPSC-Derived Vascular Smooth Muscle Cells to Promote Wound Healing

**DOI:** 10.3390/cells9040966

**Published:** 2020-04-14

**Authors:** Biraja C. Dash, Ocean Setia, Jolanta Gorecka, Hassan Peyvandi, Kaiti Duan, Lara Lopes, James Nie, Francois Berthiaume, Alan Dardik, Henry C. Hsia

**Affiliations:** 1 Section of Plastic Surgery, Department of Surgery Yale School of Medicine, Yale University, New Haven, CT 06510, USA; hassan.peyvandi@gmail.com (H.P.); kaiti.duan@yale.edu (K.D.); james.nie@yale.edu (J.N.); 2 Vascular Biology and Therapeutics Program and the Department of Surgery, Yale School of Medicine, Yale University, New Haven, CT 06510, USA; ocean.setia@yale.edu (O.S.); jolanta.gorecka@yale.edu (J.G.); laraminchillo@gmail.com (L.L.); alan.dardik@yale.edu (A.D.); 3 Department of Biomedical Engineering, Rutgers University, The State University New Jersey, Piscataway, NJ 08854, USA; fberthia@soe.rutgers.edu

**Keywords:** induced pluripotent stem cell, vascular smooth muscle cell, wound healing, paracrine factors, angiogenesis, inflammation, collagen, biomaterial

## Abstract

The application of human-induced pluripotent stem cells (hiPSCs) to generate vascular smooth muscle cells (hiPSC-VSMCs) in abundance is a promising strategy for vascular regeneration. While hiPSC-VSMCs have already been utilized for tissue-engineered vascular grafts and disease modeling, there is a lack of investigations exploring their therapeutic secretory factors. The objective of this manuscript was to understand how the biophysical property of a collagen-based scaffold dictates changes in the secretory function of hiPSC-VSMCs while developing hiPSC-VSMC-based therapy for durable regenerative wound healing. We investigated the effect of collagen fibrillar density (CFD) on hiPSC-VSMC’s paracrine secretion and cytokines via the construction of varying density of collagen scaffolds. Our study demonstrated that CFD is a key scaffold property that modulates the secretory function of hiPSC-VSMCs. This study lays the foundation for developing collagen-based scaffold materials for the delivery of hiPSC-VSMCs to promote regenerative healing through guiding paracrine signaling pathways.

## 1. Introduction

Human-induced pluripotent stem cells (hiPSCs) and their ability to differentiate into vascular smooth muscle cells (hiPSC-VSMCs) have revolutionized the field of vascular tissue engineering [1,2,3,4]. hiPSCs provide an unlimited cell source for large-scale generation of a functional and pure population of lineage- and patient-specific VSMCs without ethical concerns [3,5,6,7]. hiPSC-VSMCs exhibiting disease phenotypes have been utilized to engineer in vitro disease models [6,8,9,10]. In addition to their use in disease modeling, hiPSC-VSMCS have been exploited to build tissue-engineered vascular grafts [11,12,13,14,15]. Recently, hiPSC-VSMCS have been exploited to form microvasculature for organ tissue engineering and blood perfusion [16,17]. In one of the studies, hiPSC-VSMCs from lateral plate mesoderm lineage were shown to support human umbilical vein endothelial cells (HUVECs)-mediated angiogenesis on the Matrigel matrix via secretion of the paracrine factor neurite growth-promoting factor 2, also known as Midkine [18]. In another study, Ren et al. perfused a decellularized lung matrix with hiPSC-derived endothelial cells (ECs) and VSMCs to create a functional endothelium [19]. These preliminary reports encourage further in-depth study to explore the secretory function of hiPSC-VSMCs in extracellular-based matrix (ECM) environment.

Not only is ECM a key component of the cellular microenvironment, but its major components such as collagen, fibrin, hyaluronic acid, and elastin have widely been used to engineer biomaterials for cell delivery [20,21]. These ECM-based biomaterials dictate cellular behavior with their composition and biophysical properties [22,23,24,25,26]. Based on excellent biocompatibility properties, fibrin and collagen-based scaffolds have been designed to create therapeutic vascularization. Especially, collagen-based fibrillar scaffolds exhibit excellent biological and mechanical properties and have already been used for the therapeutic delivery of stem cells [27,28,29]. The scaffolds not only improve the survival of these cells but also enhance their paracrine activity by providing biophysical cues.

In this study, we used a collagen-based fibrillar scaffold to study (i) how biophysical properties such as collagen fibrillar density (CFD) would modulate the secretory behavior of hiPSC-VSMCs and (ii) whether this modulation of hiPSC-VSMCs has implications for regenerative wound healing. In particular, we investigated the secretory profile of important proangiogenic, tissue remodeling, and anti-inflammatory factors produced by hiPSC-VSMCs. Such factors may participate in key wound healing processes and improve the survival and therapeutic effects of hiPSC-VSMCs in vivo.

To vary the CFD, we initially used hydrated planar collagen scaffolds with different concentrations of collagen. Later, we followed a previously published report to fabricate dense fibrillar collagen (DC) scaffold by plastic compression [30,31]. The DC scaffolds in a flat and rolled configuration resulted in an increased fibrillar density and mechanical strength [32,33]. In addition, DC scaffolds in rolled configuration have been shown to create a hypoxic microenvironment by promoting cellular activation associated with increased levels of VEGF production [23,34]. The DC scaffolds have already been used to support osteoblast and fibroblast cells with enhanced cell growth and differentiation [32,35]. 

**Scheme 1:**
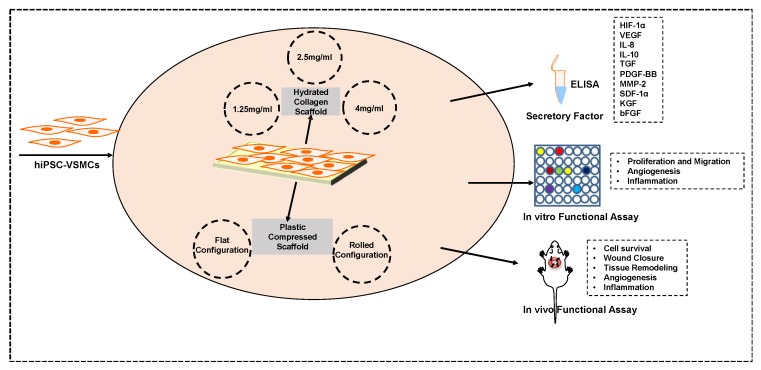
Scheme of the experimental strategy. Human induced pluripotent stem cell-derived vascular smooth muscle cells and their secretory profile in response to collagen scaffolds of varying density were characterized. In vitro functional assays of conditioned medium and in vivo testing of the cells in a splinted back wound model in nude mice were performed.

The effects of CFD on hiPSC-VSMCs have not been investigated before. In this study, we examined the effect of CFD on hiPSC-VSMC’s cell survival, production of pro-angiogenic, tissue remodeling, and anti-inflammatory factors (Scheme 1). Furthermore, the functions of these secretory factors were studied in vitro by examining the effect of conditioned medium (CM) on endothelial cells, fibroblasts, and keratinocytes, as well as in vivo by implantation of cells in a full-thickness wound-healing model. Our study suggests that an increase in CFD improves cell survival and differentially regulates hiPSC-VSMCs paracrine function. hiPSC-VSMCs produced cytokines that promote angiogenesis, immunomodulation, and wound healing. The relationship between iPSC-VSMCs’ paracrine function and CFD revealed crucial aspects of material properties that have not been thoroughly investigated before. The current study also suggests a strategy to modulate the hiPSC-VSMC secretome to improve cell-mediated regenerative healing. 

## 2. Methods

### 2.1. VSMC Differentiation and Characterization

Highly enriched VSMCs were generated in large quantities from the integration-free hiPSC line using an embryoid body (EB) method, as shown in our recently published study [6,14]. Briefly, hiPSCs were cultured under feeder-free conditions until they reached 80% confluency in a 6-well tissue culture plate (~4 days). EBs were made using ultra-low attachment 6-well plates and cultured for 5 days in an EB differentiation medium, followed by plating on gelatin-coated plates. The EBs were cultured for an additional 5 days before being transferred to Matrigel-coated plates and cultured in smooth muscle growth medium (SmGM-2) (PromoCell, Heidelberg, Germany) until 80% confluency. The cells were characterized using major smooth muscle cell markers including calponin, smooth muscle alpha-actin (SMA), SM-22α, and smooth muscle myosin heavy chain (SM-MHC) by immunofluorescence and flow cytometry.

### 2.2. Scaffold Characterization

#### 2.2.1. Cellular Scaffold Preparation

Type-I rat-tail collagen (Enzo Life Sciences, New York, USA) of an initial density of 5 mg/mL was purchased for scaffold preparation. Hydrated planar scaffolds were made using a final collagen concentration of 1.25, 2.5, and 4 mg/mL (See Supplementary Table S1). Briefly, the collagen solution (5 mg/mL) of the various amounts was mixed with 10× alpha minimum essential media (MEM) followed by neutralization with 1 M NaOH. Human iPSC-VSMCs in the SmGM-2 medium were then mixed in the neutralized gel solution. The scaffolds with and without cell controls were plated in 96-well plates. The scaffolds were then incubated at 37 °C for 30 min to solidify. The scaffolds were supplemented with 200 µL of SmGM-2 medium and were kept in culture for three days. The number of hiPSC-VSMCs embedded was 4 × 10^5^/mL. Details of the hydrated planar collagen scaffold preparation can be found in Table S1 in the supplementary information. Plastic compressed collagen scaffolds in flat and rolled configurations were fabricated using an established protocol [23]. Briefly, 4 mL of type I rat-tail collagen solution (5 mg/mL) was mixed with 500 µL of 10× alpha MEM, and 90 µL of 1 M NaOH was added to neutralize the solution followed by addition of 500 µL of the cell suspension to make the total volume 5 mL. In the case of acellular scaffold, 500 µL of the medium was used instead of cell suspension. The mixture was then poured into a mold and allowed to incubate for 30 min at room temperature until they formed the gel. The total number of hiPSC-VSMCs embedded was 2 × 10^6^. The collagen was removed from the mold and placed on the top of a nylon mesh which was supported by a blotting paper. The collagen sheet was then produced by plastic compression, as previously described [23,31], by applying an unconfined compression of 1 kPa for 5 min at room temperature. During this process, the collagen gels of an approximate size of 3.6 mm were compressed into a flat sheet of around 20–30 µm and rolled following a previously published protocol [23,31]. The flat sheets containing hiPSC-VSMCs were then rolled manually using forceps. The flat and rolled scaffolds were cultured in 3 mL of SmGM-2 medium for 72 h in a 6-well plate and then unrolled for further characterization, such as cell viability, proliferation, and phenotype. 

#### 2.2.2. AlamarBlue Assay

Scaffolds of varying densities (1.25, 2.5 and 4 mg/mL) with iPSC-VSMCs were made in a 96-well plate and tested for cell viability using an alamarBlue assay 3 days post-creation. At each time point, the cells were incubated with 100 µL of alamarBlue reagent (Thermofisher) for 2 h at 37 °C in a cell culture incubator. The color change was subsequently determined using absorbance at 570 nm and/or fluorescence (excitation: 540 and emission: 590 nm).

#### 2.2.3. Cell Proliferation, Apoptosis, and Hypoxia

DC scaffolds in flat and rolled configurations were immune-stained using anti-Ki67, anti-caspase-3, and anti-HIF-1α primary antibodies (1:200 dilutions). Caveolin-1 and dapi were used as counterstains for cell membranes and nuclei, respectively. The total number of cells was determined by manually counting the number of Ki67, caspase-3, and HIF-1α positive cells expressed as a percentage of total cells. Quantification was based on an average of five different fields of view per sample. 

#### 2.2.4. Phenotype Characterization

Cells embedded both in rolled and flat DC scaffolds were stained using anti-calponin, anti-SMA, anti-SM-22α, and anti-SM-MHC primary antibodies. Dapi was used as a counterstain, and the percentage of positive cells was determined as previously described. 

#### 2.2.5. ELISA

Conditioned media collected after a 72 h scaffold incubation were used to perform qualitative and/or quantitative ELISA to determine the concentration of various cytokines, paracrine factors, and proteases secreted by iPSC-VSMCs, except for HIF-1α, where scaffold lysate was used. Quantitative ELISA for VEGF (R&D, Minneapolis, MN, USA) was performed based on the manufacturer’s instruction. For qualitative ELISA, the microwells of a 96-well ELISA plate (NUNC MaxiSorp™) were coated by pipetting 100 μL of the protein samples. The plate was incubated overnight at 4 °C, followed by washing three times with PBST (PBS + 0.05% Tween-20) and blocking with 5% BSA for 1 hr at 37 °C. The plate was then washed three times and was incubated with primary antibodies (1:1500) overnight at 4 °C. This was followed by washing three times with PBST and incubation with secondary antibodies conjugated with horseradish peroxidase (1:2500) for 2 hr. After washing three times with PBST, 100 µL of TMB substrate solution (Cell Signaling Technology, Danvers, MA, USA) was dispensed into each microwell and incubated for 25 min at room temperature. Then, 100 μL of stop solution (Cell Signaling Technology) was added to each microwell, and absorbance was measured at 450 nm on a plate reader. Factors tested included: hypoxia inducing factor (HIF)-1α, VEGF, basic fibroblast growth factor (bFGF), matrix metalloproteinase (MMP)-2, interleukin (IL)-8, IL-10, transforming growth factor (TGF)-β, platelet-derived growth factor (PDGF)-AA, angiopoietin (ANG)-1, keratinocyte growth factor (KGF), and stromal cell-derived factor (SDF)-1α. The detailed list of antibodies with manufacturer information can be found in the supplementary information (Tables S2 and S3). 

#### 2.2.6. Cell Proliferation and Migration Assay

Human umbilical vein endothelial cells (HUVECs), primary human skin fibroblasts, and keratinocytes were grown in their respective culture medium (see Supplementary Information Table S4) until 80% confluency. The cells were then dissociated using TrypLE and were used for subcultures and cell proliferation and migration assays. Cell proliferation assays for HUVECs, fibroblasts, and keratinocytes were performed in a 96-well plate. Briefly, 6000 cells were plated on a 96-well plate and cultured for 1 day before incubating in conditioned medium from flat and rolled DC scaffolds for 24 h, as well as control medium from the acellular scaffold. For migration, trans-well assays were performed using the aforementioned cell lines. Briefly, 1 × 10^4^ cells were seeded on the top layer of the trans-wells (8 µm pore size trans-wells, Falcon) and incubated for 10 min in cell culture incubators. The trans-wells were then placed in 24-well plates, and 600 µL of respective growth medium with conditioned medium (CM) was added to the wells touching the inner side of the trans-well. The trans-wells were then kept in the cell culture incubator for 4 h. This was followed by fixing with 100% cold methanol and gently removing the cells from the top layer. Trans-wells were washed and stained using dapi. The number of cells migrated to the inner side of the membrane was then quantified by counting the number of dapi-stained nuclei from 5 different fields of view. 

#### 2.2.7. In Vitro Angiogenesis Assay

The ability of hiPSC-VSMC-CM from DC scaffolds to induce angiogenesis was measured using an in vitro angiogenesis assay as described [36]. HUVECs were cultured until they were 80% confluent in EGM-2 medium. One hundred microliters per well of Matrigel (Corning Life Sciences) was spread evenly over the wells of a 96-well plate. The plates were incubated for 30 min at 37 °C to allow the Matrigel to solidify. HUVECs (2 × 10^5^/mL) were resuspended in EGM-2 basal medium, and 100 µL of this along with 100 µL of conditioned medium was seeded in each well (*n* = 6). After 16 h of incubation of the plate at 37 °C, the floating cells were removed and the plates were washed (2×) with PBS and fixed using PFA for bright field imaging. Images from 5 different fields/well were acquired and the number of nodes per field was counted. 

#### 2.2.8. ICAM-1 Expression Analysis

The ability of CM from flat and rolled DC scaffolds to reduce inflammation was characterized using HUVECS. TNFα was used to induce inflammation on endothelial cells as described [37]. Briefly, 6000 cells/well were seeded and cultured for one day before incubation with 50 ng/mL of TNFα (Peprotech). Cells were then treated with conditioned medium from flat and rolled scaffolds, as well as medium from acellular scaffolds to serve as the control. Cells with and without TNFα were used as positive and negative control groups, respectively. The cells were fixed after a 24 h incubation and stained for ICAM-1. The percentage of ICAM-1 positive HUVECs was determined for all the groups as previously described.

### 2.3. Animal Studies

#### 2.3.1. Splinted Acute Back Wound Model and Grafting

All the animal experiments were approved by Yale’s Institutional Animal Care & Use Committee and performed following the National Institutes of Health guide for the care and use of laboratory animals. Nude mice (male, 10–12 weeks) were used to create splinted excisional wounds as previously described [23,38,39]. Briefly, two full-thickness wounds, 6 mm in diameter, were created on mouse dorsum, and silicone splints were sutured around the wounds to prevent contraction. Rolled DC scaffolds made using 4 mg/mL final collagen density were used for the animal experiments. Rolled DC scaffolds containing 2 × 10^6^ of hiPSC-VSMCs were used for animal experiments. The rolled DC scaffolds were cultured for 72 h in SmGM-2 medium and then unrolled to place on top of splinted back wounds. Acellular scaffolds were used as control. The wounds were then covered with Tegaderm for three days to secure the scaffolds. Digital photographs were captured at 0, 3, 7, 10, and 14 days and analyzed using Image J (NIH, Bethesda, MD) to assess wound healing. Wound closure was expressed as wound surface area compared to initial wound size, as previously described [23]. The animals were sacrificed at the end of the experiment, and wound tissues were collected for histological analysis. 

#### 2.3.2. Histology

Five micrometers of paraffin-embedded tissue sections were stained for H&E and Sirius Red. Images for each slide from 5 different high-powered fields were captured using a histological microscope. The H&E images were used to quantify epidermal and dermal thickness, while Sirius red-stained slides were used to quantify wound collagen levels. ImageJ quantification method was from already established methods [40,41]. 

#### 2.3.3. In Vvo Engraftment

The phenotype of engrafted hiPSC-VSMCs was determined by co-staining them with human leukocyte antigen (HLA) and/or SM-22α and calponin. Dapi was used to stain nuclei, and cells were counted to determine the total number of cells. The percentage of cells positive for HLA/calponin and HLA/SM-22α was quantified from the average of five different fields of view. Furthermore, slides were co-stained with either Ki67 or HIF-1α and HLA to determine cellular proliferation in vivo and their hypoxic activation. The percentage of cells positive for Ki67 and HIF-1α was determined as per the previously described method. 

#### 2.3.4. Evaluation of Vascularization

Tissue sections were stained using CD31, VEGF, and MMP-2. CD31-stained blood vessels were quantified for both treatment groups. Slides were also stained for MMP-2 and VEGF, and their co-expression in blood vessels was quantified. 

#### 2.3.5. Evaluation of Inflammation

Tissue sections were stained using IL-10, ICAM-1, and CD68 antibodies. The immune stained slides were imaged using a fluorescence microscope and the number of blood vessels stained with IL-10 and ICAM-1, and the number of cells positive for CD68 was quantified. 

## 3. Results

### 3.1. Increased Collagen Concentration Alters the Cell Proliferation and Paracrine Secretion of hiPSC-VSMCs in Hydrated Collagen Scaffolds 

**Figure 1:**
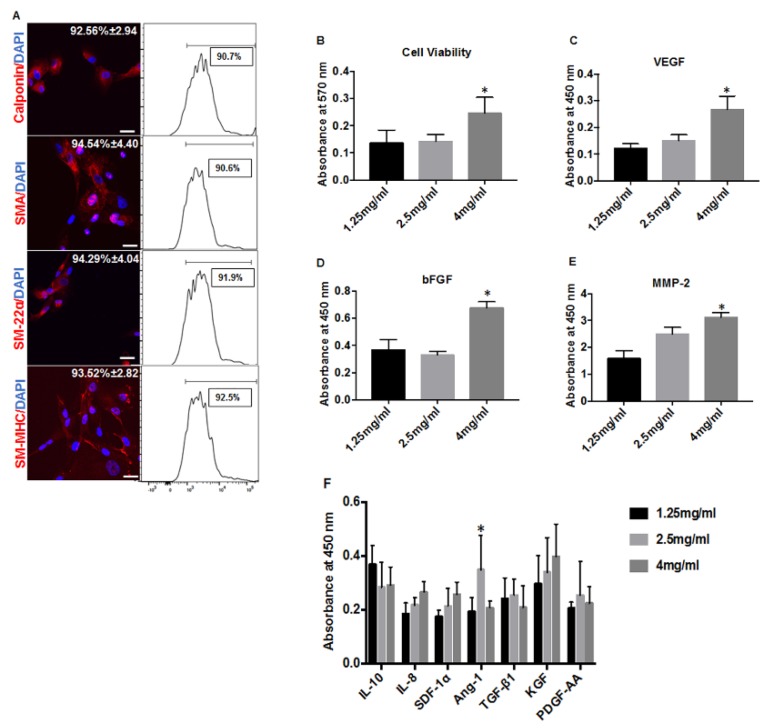
Collagen-density-dependent paracrine factor secretion of hiPSC-VSMCs in hydrated collagen scaffolds. Pure population of hiPSC-VSMCs was differentiated and characterized before embedding in the scaffolds. (**A**) Immunofluorescence images (*n* = 3) and histograms of flow cytometry analysis with markers of hiPSC-VSMCs (i) calponin, (ii) smooth muscle alpha-actin (SMA), (iii) SM-22α and (iv) smooth muscle myosin heavy chain (SM-MHC). Dapi was used to stain nuclei. Scale bar measures 50 µm. (**B**) alamarBlue assay for cell viability of hiPSC-VSMC embedded in various concentrations (1.25 mg/mL, 2.5 mg/mL and 4 mg/mL) of collagen scaffolds (*n* = 5, 4 vs. 2.5 *p* < 0.02; 4 vs. 1.25 *p* < 0.009; 2.5 vs. 1.5 *p* > 0.05). Qualitative ELISA was performed on conditioned medium collected from different concentration of scaffolds for (**C**) VEGF (*n* = 6, 4 vs. 2.5 *p* < 0.0001; 4 vs. 1.25 *p* < 0.0001; 2.5 vs. 1.5 *p* > 0.05), (**D**) bFGF (*n* = 6, 4 vs. 2.5 *p* < 0.0001; 4 vs. 1.25 *p* < 0.0001; 2.5 vs. 1.5 *p* > 0.05), (**E**) MMP-2 (*n* = 6, 4 vs. 2.5 *p* < 0.005; 4 vs. 1.25 *p* < 0.0001; 2.5 vs. 1.5 *p* > 0.0003). Statistical analysis was performed using one-way ANOVA. (**F**) IL-10, IL-8, SDF-1α, TGFβ, KGF, PDGF (*n* = 6, *p* > 0.05) and Ang-1 (*n* = 6, 2.5 vs. 4 *p* < 0.006; 2.5 vs. 1.5 *p* < 0.003; 1.25 vs. 4 *p* > 0.05). Statistical analysis was performed using two-away ANOVA. * denotes statistical significance.

Integration-free hiPSCs derived from neonatal fibroblasts were used for differentiation to VSMCs according to the previous protocol [6]. The hiPSC-VSMCs, when stained with calponin (92.56% ± 2.94), SMA (94.54% ± 4.40), SM-22α (94.29% ± 4.04), and SM-MHC (93.52% ± 2.82), were found to be more than 90% positive for each of these major VSMC markers (*n* = 3). The purity of the differentiated cells was further confirmed by flow cytometry analysis (Figure 1A). Proliferation of hiPSC-VSMCs embedded in different scaffold concentration, obtained using alamarBlue assay, revealed a significantly increased proliferation rate in 4 mg/mL scaffold group, compared to that of 2.5 (*n* = 5, *p* < 0.02) and 1.25 mg/mL (*n* = 5, *p* < 0.009) groups (Figure 1B). There was no significant difference in cell proliferation between 2.5 and 1.25 mg/mL scaffolds (Figure 1B).

Qualitative analysis of several growth factors was performed on the CM after incubation of varying concentrations of scaffolds and revealed a significantly enhanced VEGF secretion by day 3 (Figure 1C) in the 4 mg/mL scaffold group, compared to 1.25 mg/mL (*n* = 6, *p* < 0.0001) and 2.5 mg/mL (*n* = 6, *p* < 0.0001). There was no significant difference between 2.5 mg/mL and 1.25 mg/mL scaffold groups. bFGF (Figure 1D) was significantly higher in 4 mg/mL scaffolds than 2.5 mg/mL scaffolds (*n* = 6, *p* < 0.0001) and 1.25 mg/mL group (*n* = 6, *p* < 0.0001). MMP-2 secretion increased with increasing collagen density (Figure 1E), where 4 mg/mL had much higher secretion than 2.5 (*n* = 6, *p* < 0.005) and 1.25 mg/mL of scaffold groups (*n* = 6, *p* < 0.0001), and 2.5 mg/mL showed an increased expression compared to 1.25 mg/mL scaffolds (*n* = 6, *p* < 0.0003). Ang-1 (Figure 1F) was significantly upregulated in 2.5 mg/mL scaffolds compared to both 1.25 mg/mL (*n* = 6, *p* < 0.003) and 4 mg/mL groups (*n* = 6, *p* < 0.006). There was no significance difference between 1.25 mg/mL and 4 mg/mL scaffolds. Concentration of paracrine growth factors including IL-10, IL-8, SDF-1α, TGFβ, KGF, and PDGF-AA were also measured (Figure 1F) but did not reveal any significant differences between the various densities of scaffolds (*n* = 6, *p* > 0.05). 

### 3.2. A Rolled DC Scaffold Differentially Modulates iPSC-VSMCs’ Secretory Function

**Figure 2:**
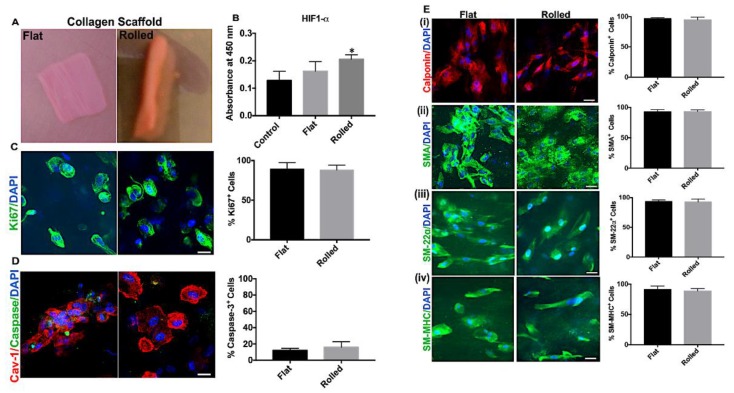
A dense fibrillar collagen (DC) scaffold in rolled configuration produces a hypoxic environment and maintains cell viability and morphology of hiPSC-VSMCs. Type I collagen was used to build DC scaffolds of flat and rolled configuration. The hiPSC-VSMCs embedded were characterized for cell viability and phenotype. (**A**) Gross images of flat and rolled scaffold embedding hiPSC-VSMCs in culture. The scaffolds were in the rolled configuration for 72 h to create a hypoxic environment. (**B**) Qualitative ELISA analysis showing the expression of HIF-1α in the flat and rolled cellular scaffolds. The cellular hydrated collagen (4 mg/mL) and flat scaffolds were used as a control (*n* = 8, Rolled vs. Control *p* < 0.0001; Rolled vs. Flat *p* < 0.03; Flat vs. Control *p* > 0.05). (**C**) Ki67 immuno-stained flat and rolled scaffolds and the graph representing % Ki67 positive cells after three days of culture (*n* = 4, *p* > 0.05). (**D**) Caspase-3 immuno-stained flat and rolled scaffolds and the graph representing % caspase-3 positive cells after three days of culture (*n* = 4, *p* > 0.05). Caveolin-1 was used to stain the cell membrane and dapi for nuclei. Phenotype characterization of hiPSC-VSMCs cultured in flat and rolled configuration for three days were performed by immunostaining with major VSMC markers (**Ei**) calponin (*n* = 4, *p* > 0.05), (**Eii**) SMA (*n* = 4, *p* > 0.05), (**Eiii**) SM-22α (*n* = 4, *p* > 0.05), and (**Eiv**) SM-MHC (*n* = 4, *p* > 0.05). Dapi was used to stain nuclei. Scale bar measures 50 µm. Statistical analysis was performed using one-way ANOVA and student *t*-test. * denotes statistical significance.

In another setting, collagen scaffolds containing the final concentration of 4 mg/mL collagen in 5 mL of total gel solution were plastically compressed to fabricate biomimetic DC scaffolds (Figure 2A). The plastic compression removed approximately 80% of the water content, making the DC scaffold highly dense (~20 mg/mL) and led to an inherent increase in cell density (~2 × 10^6^ cells/mL). The rolled scaffolds kept in the medium maintained their rolling configuration throughout the culture (3 days) and did not disrupt the basic collagen scaffold structure. hiPSC-VSMCs in rolled scaffolds were tested for cell viability, morphology, HIF-1α, and secretory factors. Flat scaffolds were used as control. 

**Figure 3:**
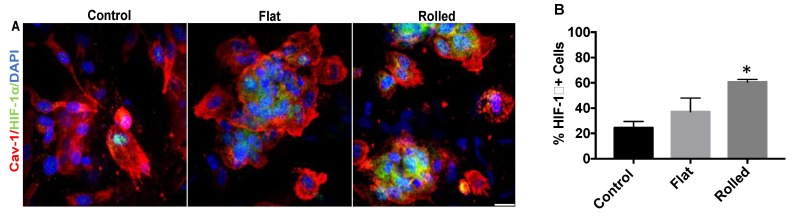
Immunofluorescence characterization of HIF-1α expression in DC scaffolds. (**A**) Immunofluorescence images showing expression of HIF-1α (Green) in control (4 mg/mL hydrated scaffold), flat, and rolled DC scaffolds. Caveolin-1 (Red) was used to stain the cell membrane and dapi (Blue) for nuclei. Scale bar measures 20 µm. (**B**) The graph represents % HIF-1α positive cells. Statistical analysis was performed using one-way ANOVA and Student *t*-test (*n* = 3, Rolled vs. Control *p* < 0.002; Rolled vs. Flat *p* < 0.02; Flat vs. Control *p* > 0.05). * denotes statistical significance.

**Figure 4:**
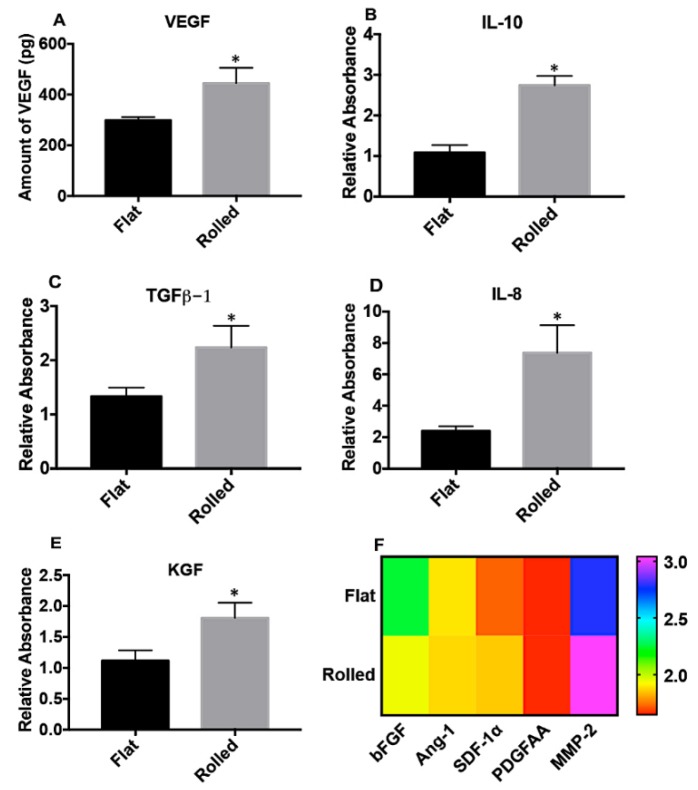
A DC scaffold in rolled configuration modulates the paracrine function of hiPSC-VSMCs. hiPSC-VSMCs embedded in DC scaffolds in a rolled configuration for 72 h exhibited hypoxia and affected the cells’ secretory function. Qualitative ELISA was performed on conditioned media collected from flat and rolled scaffolds for (**A**) VEGF (*n* = 6, Rolled vs. Flat *p* < 0.003), (**B**) IL-10 (*n* = 6, Rolled vs. Flat *p* < 0.0001), (**C**) TGFβ (*n* = 6, Rolled vs. Flat *p* < 0.004), (**D**) IL-8 (*n* = 6, Rolled vs. Flat *p* < 0.0008), and (**E**) KGF (*n* = 6, Rolled vs. Flat *p* < 0.004); (**F**) heatmap showing bFGF, SDF-1α, Ang-1, PDGF-AA, and MMP-2 (*n* = 6, Rolled vs. Flat *p* > 0.05). Conditioned medium (CM) from acellular scaffold was used as a control. Statistical analysis was performed using the student *t*-test. * denotes statistical significance.

Qualitative ELISA performed on the lysates from rolled scaffolds showed a significantly increased expression of HIF-1α compared to control planar scaffolds (*n* = 8, *p* < 0.0001) and flat scaffolds (*n* = 8, *p* < 0.03). There was no significant difference in the expression of HIF-1α between flat scaffolds and control planar scaffolds (Figure 2B). Cell cytotoxicity, assessed with LDH assay, was found to be minimal in rolled DC scaffolds as compared to flat DC scaffolds (*n* = 4, *p* < 0.009) and positive controls (*n* = 4, *p* < 0.0001) (Supplementary Figure S1). The proliferation rate, as determined by Ki67 positivity, was similar, and nearly 85% in both scaffold configurations (Figure 2C, *n* = 4). Staining with caspase-3 was performed to determine any DC scaffold-mediated apoptosis, and the percentage of cells positive for caspase-3 (*n* = 4) was similarly low (approximately 10%–15%) in both flat and rolled scaffolds (Figure 2D). The effect of DC scaffolds on the VSMC phenotype was measured using major smooth muscle cell markers including calponin, SMA, SM-22α, and SM-MHC (Figure 2E(i–iv)). hiPSC-VSMCs maintained their phenotype more than 95% positive for the aforementioned markers (*n* = 4). There was no significant phenotypic difference between rolled and flat DC scaffolds (Figure 2E(i–iv)). Immunofluorescence characterization of HIF-1α in hiPSC-VSMCs embedded in various scaffold conditions showed a similar trend as the ELISA (Figure 3A). Rolled scaffolds showed ~60% of HIF-1α positive cells compared to ~24% in hydrated control scaffolds and ~37% in flat scaffolds (Figure 3B). The CM collected from rolled DC scaffolds was found to contain a significantly higher concentration of VEGF (*n* = 6, *p* < 0.003), IL-10 (*n* = 6, *p* < 0.0001), TGFβ (*n* = 6, *p* < 0.004), IL-8 (*n* = 6, *p* < 0.0008), and KGF (*n* = 6, *p* < 0.004) compared to flat DC scaffolds (Figure 4A–E). Rolled DC scaffolds showed similar levels of bFGF, Ang-1, SDF-1α, PDGF-AA, and MMP-2 compared to flat DC scaffolds (Figure 4F).

### 3.3. The Conditioned Medium from Rolled DC Scaffold Promotes Cellular Proliferation and Migration 

**Figure 5:**
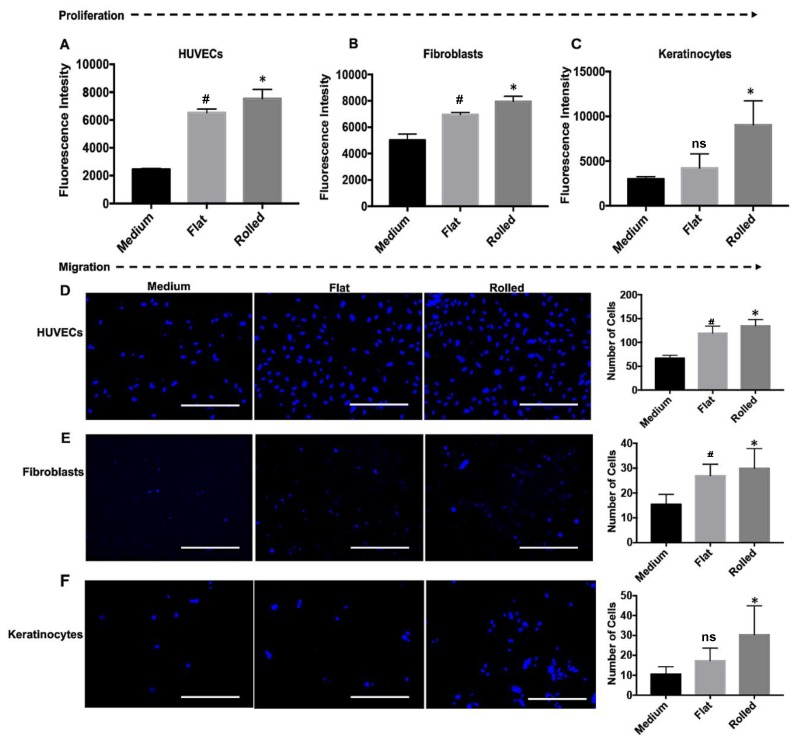
Characterization of conditioned medium from DC scaffolds for proliferation and migration of cells. The conditioned media collected from the flat and rolled scaffold culture conditions were tested for their bioactivity. An alamarBlue assay was performed to obtain proliferation data on (**A**) HUVECs (*n* = 6, Rolled vs. Flat *p* < 0.05; Rolled vs. Medium *p* < 0.0001; Flat vs. Medium *p* < 0.0001), (**B**) primary human skin fibroblasts (*n* = 6, Rolled vs. Flat *p* < 0.05; Rolled vs. Medium *p* < 0.0005; Flat vs. Medium *p* < 0.001), and (**C**) primary human skin keratinocytes (*n* = 6, Rolled vs. Flat *p* < 0.001; Rolled vs. Medium *p* < 0.0002; Flat vs. Medium *p* > 0.05) 24 h after the addition of CM to their culture media. Eight micrometer pore size trans-wells were used to determine migration of (**D**) HUVECs (*n* = 4, Rolled vs. Flat *p* > 0.05; Rolled vs. Medium *p* < 0.0001; Flat vs. Medium *p* < 0.0005), (**E**) primary human skin fibroblasts (*n* = 4, Rolled vs. Flat *p* > 0.05; Rolled vs. Medium *p* < 0.02; Flat vs. Medium *p* < 0.05), and (**F**) primary human skin keratinocytes (*n* = 4, Rolled vs. Flat *p* > 0.05; Rolled vs. Medium *p* < 0.04; Flat vs. Medium *p* > 0.05) in response to the CM over 4 h of incubation time. The cells migrated were stained with dapi and counted to obtain the total number of cells migrated. The scale bar represents 200 µm. SmGM-2 medium from the acellular scaffold was used as a control. Statistical analysis was performed using one-way ANOVA. * and **^#^** denote statistical significance, and ns represents no significance.

Conditioned media obtained from rolled and flat scaffolds were tested for their in vitro functionality and effect on important wound healing processes, such as proliferation and migration of endothelial, fibroblast, and keratinocyte cells (Figure 5). The CM acellular scaffolds were used as control. HUVEC displayed an enhanced level of proliferation when treated with CM from both flat (*n* = 6, *p* < 0.0001) and rolled scaffolds (*n* = 6, *p* < 0.0001) compared to control acellular scaffolds, with rolled scaffolds having a larger effect when compared to flat scaffolds (Figure 5A, *n* = 6, *p* < 0.05). Fibroblasts (Figure 5B) showed a similar response to CM from flat (*n* = 6, *p* < 0.001) and rolled scaffolds (*n* = 6, *p* < 0.0005) compared to the control acellular scaffolds, with rolled scaffolds having a larger effect than flat scaffolds (*n* = 6, *p* < 0.05). Keratinocyte (Figure 5C) proliferation was only significantly increased in response to CM from rolled scaffolds, as compared to the control group (*n* = 6, *p* < 0.0002) and flat scaffolds (*n* = 6, *p* < 0.001), with no difference between flat scaffolds and control. Conditioned media from both rolled and flat scaffolds enhanced the migration of HUVECs, human skin fibroblasts, and keratinocytes (Figure 5D–F). Rolled scaffold CM had a larger effect on migration of HUVECs (*n* = 4, *p* < 0.0001), fibroblasts (*n* = 4, *p* < 0.02), and keratinocytes (*n* = 4, *p* < 0.04), compared to the control (Figure 5D–F). Although we observed a significant increase in migration of HUVECs (*n* = 4, *p* < 0.0005) and fibroblasts (*n* = 4, *p*< 0.05) after treatment of flat scaffold CM, this effect was significantly lower than that of rolled scaffolds (Figure 5D,E). The CM from flat scaffolds did not affect keratinocyte migration (Figure 5E). 

### 3.4. Conditioned Medium from Rolled DC Scaffold Has a Pro-Angiogenic and Anti-Inflammatory Effect 

**Figure 6:**
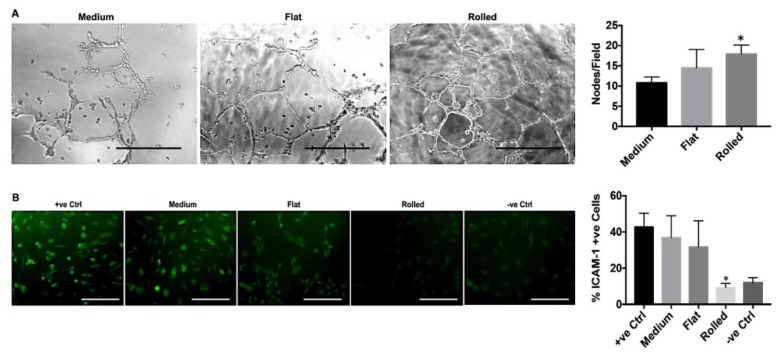
Conditioned medium from DC scaffold in rolled configuration enhances angiogenesis and reduces TNFα-mediated inflammation on endothelial cells. The conditioned media from the flat and rolled scaffold culture conditions collected were tested for their pro-angiogenic and anti-inflammatory potentials. Angiogenesis assay was performed using Matrigel and HUVECs. (**A**) Brightfield images showing tube formation of HUVECs in response to CM from flat and rolled scaffolds after 16 h of incubation. SmGM-2 from acellular scaffold was used as a control medium. The scale bar represents 200 µm. The graph represents number of nodes/field (*n* = 4, Rolled vs. Flat *p* > 0.05; Rolled vs. Medium *p* < 0.03; Flat vs. Medium *p* > 0.05). TNFα of 50 ng/mL was used to induce inflammation in HUVECs and then were treated with conditioned media from flat and rolled scaffolds. Control groups were TNFα alone, no TNFα and SmGM-2 from an acellular scaffold. The expression of ICAM-1 was detected and quantified. (**B**) Immunostaining of HUVECs with ICAM-1 and the representative graph shows the percentage of cells positive for ICAM-1 (*n* = 4, Positive control vs. Negative control *p* < 0.0003; Rolled vs. Positive control <0.002; Medium control vs. Flat *p* > 0.05). Statistical analysis was performed using one-way ANOVA. * denotes statistical significance.

Pro-angiogenic and anti-inflammatory effects of rolled and flat scaffold CM were tested using a well-established angiogenesis assay [36] and TNFα-induced ICAM-1 expression method [37], respectively. The angiogenesis assay showed an increase in the number of nodes/field in response to CM from rolled scaffolds (17.9 ± 1.115), compared to control (10.8 ± 0.7257) and flat scaffolds (14.5 ± 2.281). There was a significant difference between rolled scaffold and control groups (*n* = 4, *p* < 0.03), with no significant difference in the number of nodes/field between flat scaffold and control (Figure 6A). To induce inflammation and examine the immunomodulatory effect of the conditioned medium, HUVECs were treated with TNFα, and ICAM-1 expression was measured (Figure 6B). Groups included TNFα-only as a positive control, TNFα and cell-free medium, TNFα and flat scaffold CM, TNFα and rolled scaffold CM, and no TNFα treatment as a negative control. The positive control with TNFα showed a greater level of ICAM-1 expression on HUVECs compared to the negative control (*n* = 4, *p* < 0.003). Control medium and flat scaffold CM displayed a similar expression level of ICAM-1 as the positive control (*n* = 4, *p* > 0.05). However, CM from the rolled scaffold group showed a significantly reduced amount of ICAM-1 expression (*n* = 4, *p* < 0.002), and the level was found to be similar to that of the negative control group (Figure 6B).

### 3.5. hIPSC-VSMCs in a Rolled DC Scaffold Enhances Wound Closure and Tissue Remodeling

**Figure 7:**
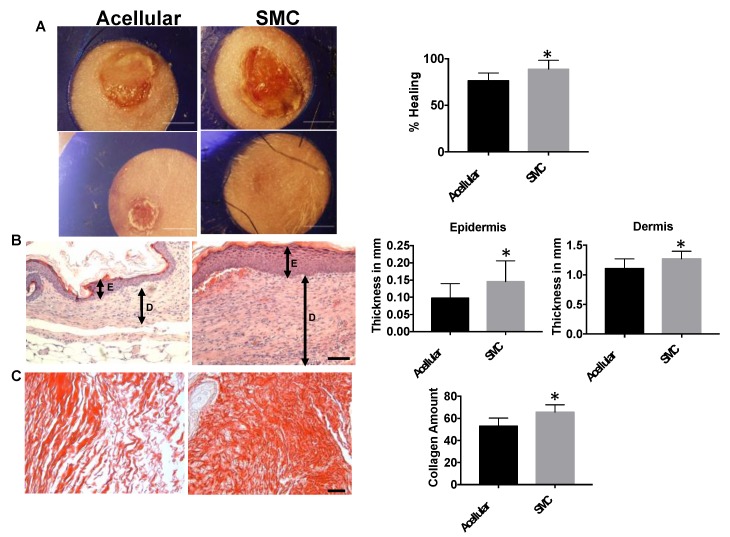
hiPSC-VSMCs in a rolled DC scaffold promotes wound healing and in vivo cell survival. The hiPSC-VSMCs cultured in rolled scaffolds for 72 h were implanted in splinted back wound in nude mice and were observed for 14 days. Acellular scaffolds were used as control groups. (**A**) Gross morphology showing day 0 and 10 wound closure. The graph represents percentage wound closure of the treatment groups acellular and hiPSC-VSMC-based scaffolds (*n* = 9, hiPSC-VSMC vs. Acellular *p* < 0.01). (**B**) H&E images of regenerated skin showing epidermis and dermis of wounds treated with acellular and hiPSC-VSMC scaffolds. The graphs represent the thickness of the epidermis (*n* = 9, hiPSC-VSMC vs. Acellular *p* < 0.006) and dermis (*n* = 9, hiPSC-VSMC vs. Acellular *p* < 0.05). (**C**) Sirius Red staining of wound tissue showing collagen (Red). The graph represents the amount of collagen in the wounds treated with acellular and hiPSC-VSMC scaffolds (*n* = 7, hiPSC-VSMC vs. Acellular *p* < 0.006). Statistical significance was determined using the Student *t*-test. In vivo cell survival was characterized in the explanted tissue treated with hiPSC-VSMC scaffolds. * denotes statistical significance.

Rolled DC scaffold containing hiPSC-VSMCs were implanted on full-thickness wounds in a nude mouse, splinted, back wound model, and their wound healing potential was compared to acellular scaffolds. Percentage wound closure was measured on day 10 and compared to day 0 wound area (Figure 7A). By day 10, the wound closure was found to be larger in hiPSC-VSMC scaffold treated wounds (88.9 ± 3.198), as compared to (76.48 ± 2.73) acellular scaffold treated group (*n* = 9, *p* < 0.01). By day 14, both acellular and hiPSC-VSMC-treated wounds showed complete wound closure. Data for all the time points can be found in Supplementary Figure S2. The epidermis was found to be significantly thicker in the hiPSC-VSMC-treated group (0.1454 mm ± 0.02019) compared to acellular scaffolds (0.09783 mm ± 0.01391) (Figure 7B, *n* = 9, *p* < 0.006). Similarly, the dermal thickness was found to be higher in the hiPSC-VSMC group (1.27 mm ± 0.04254), compared to the control acellular (1.106 mm ± 0.05392) group (Figure 7B, *n* = 9, *p* < 0.05). Lastly, wounds treated with hiPSC-VSMC contained elevated amount collagen (Figure 7C) (65.55 ± 2.509) as compared to the control (52.88 ± 2.778) group (*n* = 7, *p* < 0.02).

### 3.6. Hypoxia-Induced Activation of iPSC-VSMC Promotes Their Survival In Vivo

**Figure 8:**
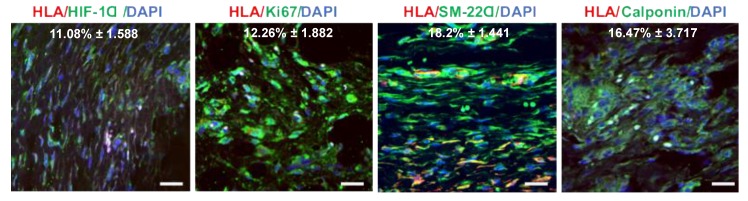
In vivo viability and phenotype of hiPSC-VSMCs in the wound microenvironment. Immunofluorescence of explanted tissue from hiPSC-VSMC-treated groups using (i) HLA/calponin, (ii) HLA/SM-22α, (iii) HLA/Ki67, and (iv) HLA/HIF-1α. Dapi was used to stain nuclei. Scale bar represents 50 µm. Data (*n* = 4) are presented as mean ± SEM.

Implanted hiPSC-VSMC scaffolds harvested after day 14 were stained with calponin, SM-22α, Ki67, and HIF-1α (*n* = 4). HLA was used to determine cells of human origin. Cells stained for HLA^+^/calponin^+^ were quantified and found to be 18%–25% of the total cell population (Figure 8). HLA^+^/SM-22α^+^ cells were found to be in a similar range (Figure 8). The percentage of cells dually positive for HLA^+^/Ki67^+^ (Figure 8) and HLA^+^/HIF-1α^+^ were found to be 10%–16% of the total cell population (Figure 8).

### 3.7. hIPSC-VSMC Secretory Factor-Mediated Vascularization and Immunomodulation in the Wound Microenvironment

**Figure 9:**
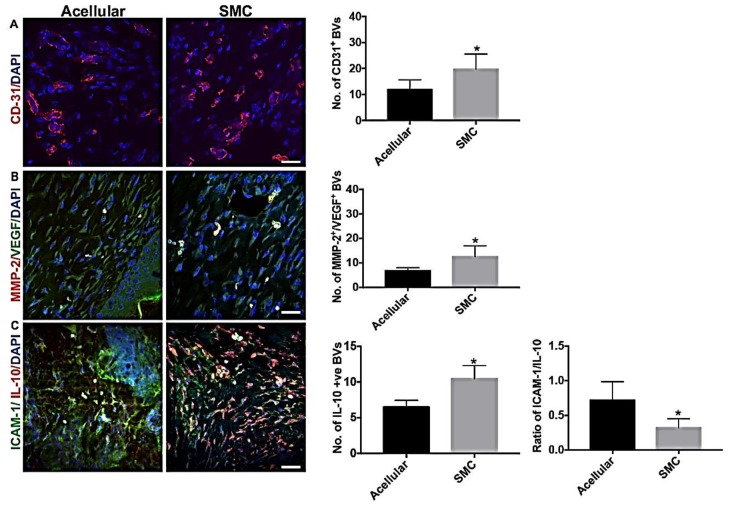
hiPSC-VSMC-mediated angiogenesis and anti-inflammation of blood vessels in vivo. In vivo, angiogenesis and the anti-inflammatory role of hiPSC-VSMCs were characterized. Immunostaining of the wound site, with (**A**) CD31 detecting the blood vessels and the graph showing the number of blood vessels for wounds treated with acellular and hiPSC-VSMC scaffolds, (*n* = 7, hiPSC-VSMC vs. Acellular *p* < 0.02). (**B**) VEGF and MMP-2 showing co-expression of these factors in the blood vessels, and the graph represents the number of blood vessels positive for both VEGF and MMP-2 (*n* = 4, hiPSC-VSMC vs. Acellular *p* < 0.05) and (**C**) IL-10 and ICAM-1 showing the level of these factors in the blood vessels and the graph represent the level of IL-10 (*n* = 4, hiPSC-VSMC vs. Acellular *p* < 0.009) and the ratio of expression of ICAM-1/IL-10 (*n* = 4, hiPSC-VSMC vs. Acellular *p* < 0.05). Dapi was used to stain nuclei. Scale bar represents 50 µm. Statistical analysis was performed using the Student *t*-test. * denotes statistical significance.

**Figure 10:**
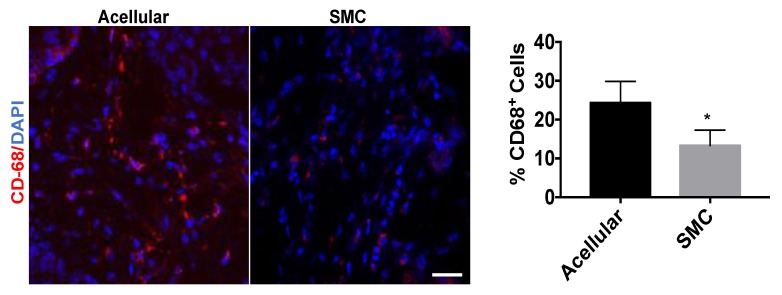
hiPSC-VSMC-mediated modulation of macrophages in vivo. In vivo, the anti-inflammatory role of hiPSC-VSMCs on macrophages was characterized. Immunostaining of the wound site with CD68 detecting the macrophages and the graph showing the number of macrophages for wounds treated with acellular and hiPSC-VSMC scaffolds, (*n* = 4, hiPSC-VSMC vs. Acellular *p* < 0.02). Dapi was used to stain nuclei. Scale bar represents 50 µm. Statistical analysis was performed using the Student *t*-test. * denotes statistical significance.

Explanted wound tissue was tested for the endogenous level of angiogenesis and endothelial cell inflammation. Angiogenesis (Figure 9A) was significantly upregulated in the hiPSC-VSMC-treated group 19.74 ± 2.21 compared to acellular scaffolds 11.89 ± 1.436 (*n* = 7, *p* < 0.02), as determined by the number of CD31+ blood vessels. Wound tissue also showed co-expression of MMP-2^+^/VEGF^+^ blood vessels (Figure 9B). The number of MMP-2^+^/VEGF^+^ co-expressing blood vessels was significantly higher in the hiPSC-VSMC-treated group (12.5 ± 2.235), compared to the acellular treatment group (6.85 ± 0.5795) (*n* = 4, *p* < 0.05). IL-10 and ICAM-1 levels were examined to demonstrate the anti-inflammatory activity of hiPSC-VSMC scaffolds in vivo (Figure 9C). An enhanced IL-10 expression was observed in the hiPSC-VSMC treated group 10.46 ± 0.9216 compared to acellular scaffolds 6.488 ± 0.471 (*n* = 4, *p* < 0.009). We observed minimal expression of IL-10 in the blood vessels of control groups. The ICAM-1 to IL-10 ratio was significantly reduced in the treatment group (0.3212 ± 0.06553) as compared to control 0.7192 ± 0.1332 (*n* = 4, *p* < 0.04). hiPSC-VSMC-mediated immunomodulation was further validated with CD68 immunofluorescence staining for inflammatory cells (Figure 10). CD68 immunostaining data showed that overall macrophages are fewer for the hiPSC-VSMC treated group compared to the control acellular groups by day 14 (*n* = 4, *p* < 0.02).

## 4. Discussion

The recent developments in reprogramming and differentiation techniques have revolutionized the field of iPSC-VSMC and their regenerative therapy [2,42,43]. While the integration-free reprogramming techniques pave the path to generating clinical-grade hiPSC-VSMCs, improved differentiation techniques bring scalability and efficiency to their production. hiPSC-VSMCs from lateral plate mesoderm lineage has lately been shown to support the formation of robust microvasculature via secreting proangiogenic growth factors [43]. Our EB-based differentiation method has earlier been shown to produce a large scale of VSMCs resembling that from lateral plate mesoderm and forms the base of our current study [6].

The paracrine secretion and survival of stem cells are always the subject of research in the field of regenerative healing and biomaterial design [28]. Functional biomaterials using ECM-based biopolymers such as collagen have been engineered to provide stem cells a niche and mechanical and biochemical cues to promote the secretion of proangiogenic and/or anti-inflammatory paracrine factors [44,45,46]. Thus, understanding the cell–matrix interaction and their underlying signaling pathways is of paramount importance while developing an efficient cell delivery system. In this study, we use type-I collagen-based fibrillar scaffold with tunable fibrillar density to understand the effect of collagen density on cell survival and paracrine secretion of iPSC-VSMCs. The hydrated collagen scaffolds made using 1.25, 2.5, and 4 mg/mL represent a serial increase in the collagen fibrillar density and affected the secretion of growth factors in hiPSC-VSMCs. The current study showed that the hydrated collagen scaffolds enhanced the secretion of key proangiogenic factors VEGF, bFGF, and MMP-2 with an increase in collagen density. However, this incremental change in hydrated collagen density did not affect the secretion of anti-inflammatory cytokines and tissue remodeling growth factors such as IL-8, IL-10, SDF-1α, TGFβ, PDGF, Ang-1, and KGF.

We further examined the effect of a highly dense collagen scaffold on hiPSC-VSMCs in a plastic compressed collagen scaffold. We followed an earlier published method to create the plastic compressed DC scaffolds using 4 mg/mL final concentration of collagen [23]. The method removes the water content of the collagen scaffolds and increases their collagen fibrillar (~20 mg/mL) and cell density (2 × 10^6^/mL) in addition to mechanical strength [30]. The flat sheet of the DC scaffold when rolled increases the density and creates a hypoxic environment [23]. While our earlier study already established that rolled scaffolds create a hypoxic environment by reducing the oxygen tension, we here demonstrated at the molecular level the upregulation of HIF-1α, a key regulator of the hypoxia response, in hiPSC-VSMCs as a result of rolling the DC scaffolds. Several previous studies showed DC scaffolds negatively influencing cellular viability, whereas our current study reported optimal cell growth and survival with an elevated number of proliferative hiPSC-VSMCs. Besides, the DC scaffolds not only maintained the phenotype of hiPSC-VSMCs but also promoted it as evident from an increase in the expression of VSMC markers from approximately 90% in 2D cell culture to 95% in the scaffolds.

By profiling cytokines relevant to wound healing, our study showed that the rolled DC scaffold provides a unique microenvironment capable of enhancing hiPSC-VSMCs paracrine secretions and showed a different pattern of secretion compared to the hydrated collagen scaffolds. In the case of rolled DC scaffolds, not only VEGF but also known pro-wound healing cytokines and growth factors IL-8, IL-10, TGFβ, and KGF were highly expressed. The concentration of other growth factors including bFGF, PDGF-BB, SDF-1α, Ang-1, and MMP-2 was significantly enhanced in both rolled and flat DC scaffolds compared to controls. Mechanical forces and hypoxia have been recognized as critical factors determining various cellular functions [47,48,49,50,51,52,53] and can be evident from an increased level of secretory factors from flat DC scaffolds. However, the differential secretory function of hiPSC-VSMCs in rolled DC scaffolds can be attributed to a combination of hypoxia and mechanical force at the micro-scale level.

Studying the paracrine function of hiPSC-VSMCs has a profound implication for understanding how these cells transplanted in vivo can accelerate wound closure and facilitate regenerative healing. Although the CM from flat DC scaffolds showed significant bioactivity compared to the control, the secretion of proangiogenic growth factors from the rolled DC scaffold such as VEGF, bFGF, KGF, TGFβ, PDGF-BB, SDF-1α, Ang-1, IL-8, and MMP-2 was capable of wound regeneration via formation of robust micro-vasculatures and tissue remodeling. In this study, we focused on how hiPSC-VSMCs in the rolled DC scaffold will promote wound healing via secretion of the above-mentioned pro-wound healing factors. By applying the hiPSC-VSMCs in the rolled DC scaffolds to the wounds, it was demonstrated that the repertoire of hiPSC-VSMCs paracrine products, derived from rolled DC scaffolds, contained therapeutic factors that promoted tissue regeneration. This result matches the in vitro functional assays in which the pro-angiogenic, anti-inflammatory, and tissue remodeling potency of the CM were observed. The in vitro qualitative analysis of the secretory factors was correlated with both in vitro and in vivo functional assays.

One mechanism for the regenerative wound healing may be linked to the ability of these cells to survive in vivo and modulate the wound bed towards more angiogenesis and less inflammatory environment. The in vivo cell survival, proliferation, and phenotypic maintenance 14 days after implantation can be attributed to the pre-activation of hiPSC-VSMCs by hypoxia and subsequent expression of HIF-1α, as seen earlier in the case of MSCs [51]. We saw an increase in EC proliferation and migration and enhanced angiogenesis in vitro in Matrigel assay. The proangiogenic potential of hiPSC-VSMCs in rolled scaffolds can be attributed to the secretory factors VEGF, bFGF, TGFβ, PDGF-AA, SDF-1α, Ang-1, and MMP-2. In vivo, we demonstrated an increase in blood vessel formation in the treatment group and investigated a pathway responsible for this therapeutic angiogenesis. Our current study exhibited an increase in the co-expression of VEGF and MMP-2 in the newly formed blood vessels. Thus, we believe hiPSC-VSMC-mediated angiogenesis might be regulated by VEGF and MMP-2 and needs further investigation.

While increased angiogenesis is crucial to wound regeneration, most of the time, their quality is affected by the inflammatory wound environment [54]. Inflammation because of TNFα and other inflammatory regulators often leads to an increase in leaky vessels via the upregulation of adhesion molecules such as ICAM-1 [55]. In this study, we attempted to understand the role of hiPSC-VSMCs in generating a non-leaky microvasculature. The CM of rolled scaffolds secreting IL-10 was tested in an in vitro setting. The secretion of anti-inflammatory cytokine IL-10 from the rolled scaffolds reduced TNFα-mediated inflammatory responses on endothelial cells by reducing the expression of ICAM-1. In vivo, our treatment group showed higher expression of IL-10 and reduced expression of ICAM-1 in the blood vessels compared to the control. In addition to forming non-leaky vasculature, the hiPSC-VSMC treatment group reduced the number of CD68^+^ inflammatory cells in vivo. Although we do not have data showing inflammatory cells at an earlier time point, we strongly believe this anti-inflammatory effect might be combating the pro-inflammatory role of IL-8 in the wound environment. While IL-8 is widely considered as a pro-inflammatory cytokine, its role in angiogenesis is well documented [56,57,58]. One of the studies has linked the proangiogenic effect of IL-8 to the upregulation of MMP-2 and -9 [59]. Thus, we believe the secretion of IL-8 from hiPSC-VSMCs at a therapeutic level might be promoting vessel formation via MMP-2.

In addition to being pro-angiogenic, IL-8 has been attributed to keratinocyte proliferation and migration during wound healing [60]. We thus propose IL-8 along with KGF secreted from the rolled scaffolds are responsible for promoting in vitro proliferation and migration of human skin keratinocytes and resulting in a thicker epidermis in vivo. Growth factors PDGF, bFGF, and TGFβ regulate fibroblast proliferation, migration, and collagen synthesis during wound healing [61]. While our in vitro studies showed an increase in fibroblast proliferation and migration, in vivo we saw an abundant amount of collagen formation and a thicker dermis in the treatment group.

## 5. Conclusions

**Scheme 2:**
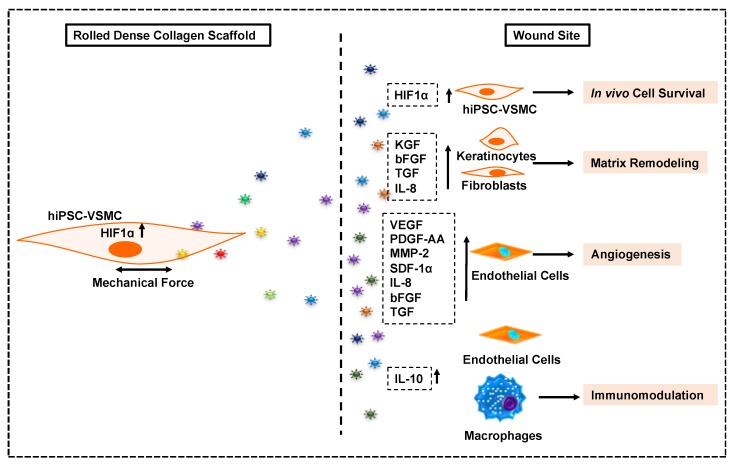
Scheme summarizing the mechanism behind regenerative wound healing. hiPSC-VSMCs in the rolled DC scaffold culture condition get activated with mechanical force and hypoxia and release secretory factors that modulate the in vivo wound environment to a regenerative one through enhancing angiogenesis and tissue remodeling and reducing inflammation.

In summary, hiPSC-VSMCs promote faster wound closure and regenerative healing as illustrated in Scheme 2. Additional studies will be conducted to further elucidate underlying signaling pathways for differential expression of paracrine factors within different scaffold conditions and hiPSC-VSMC-mediated cellular heterogeneity in the wound tissue so that the beneficial effects of the paracrine function of these cells can be controlled and maximized. In addition, in vivo studies validating the role of each of these secretory factors and revealing the underlying mechanism would be a step forward in understanding the full regenerative potential of hiPSC-VSMCs. Furthermore, a comprehensive proteomic analysis on the CM and identification of therapeutic extracellular vesicles will be important to develop cell-free therapeutics in the near future.

## Supplementary Materials

The following are available online at https://www.mdpi.com/2073-4409/9/4/966/s1: Figure S1: Cytotoxicity characterization of hypoxic collagen scaffold on iPSC-VSMC, Figure S2: Rolled DC scaffold promotes wound healing in vivo nude mice models, Table S1: Information related to fabrication of hydrated collagen scaffolds of various density, Table S2: Information related to primary antibodies, Table S3: Information related to secondary antibodies. The Supporting Information is available online and contains additional information on immunostaining, LDH assay, media composition, wound closure data, and antibodies. Data can be made available upon request.
