# Supplementary material for: A Dense Fibrillar Collagen Scaffold Differentially Modulates Secretory Function of iPSC-Derived Vascular Smooth Muscle Cells to Promote Wound Healing
Source: Cells. 2020 Apr 14;9(4):966. doi: 10.3390/cells9040966 (PMC7226960; doi:10.3390/cells9040966)
Supplement: Supplementary file 1 [file cells-09-00966-s001.pdf]

Supplementary Materials

A Dense Fibrillar Collagen Scaffold Differentially Modulates Secretory Function of iPSC-Derived Vascular Smooth Muscle Cells to Promote Wound Healing

Biraja C. Dash [1,*], Ocean Setia [2], Jolanta Gorecka [2], Hassan Peyvandi [1], Kaiti Duan [1], Lara Lopes [2], James Nie [1], Francois Berthiaume [3], Alan Dardik [2] and Henry C. Hsia [1,*]

[1] Section of Plastic Surgery, Department of Surgery Yale School of Medicine, Yale University, New Haven, CT 06510, USA; hassan.peyvandi@gmail.com (H.P.); kaiti.duan@yale.edu (K.D.); james.nie@yale.edu (J.N.)

[2] Vascular Biology and Therapeutics Program and the Department of Surgery, Yale School of Medicine, Yale University, New Haven, CT 06510, USA; ocean.setia@yale.edu (O.S.); jolanta.gorecka@yale.edu (J.G.); laraminchillo@gmail.com (L.L.); alan.dardik@yale.edu (A.D.)

[3] Department of Biomedical Engineering, Rutgers University, The State University New Jersey, Piscataway, NJ 08854, USA; fberthia@soe.rutgers.edu

* Correspondence: biraja.dash@yale.edu (B.C.D.); henry.hsia@yale.edu (H.C.H.)
Tel.: +1-203-737-2049; Fax: +1-203-785-5714 (H.C.H.)

Methods:

LDH assay: Lactate dehydrogenase assay was performed using an LDH Cytotoxicity assay kit (Peirce, Thermofisher). Briefly, the scaffolds were homogenized using mechanical force and RIPA buffer. The cell lysates were then used to detect LDH level as per manufacturer's instruction. Acellular scaffold was used as negative control, and 1x LDH was used as the positive control. The different groups that were included in this experiment were: rolled and flat scaffolds incubated for 72hr.

Immunofluorescence: Harvested scaffolds were fixed using PFA and were mounted on slides. The scaffolds were then stained with Ki67, Cav-1, caspase-3, calponin, SMA, SM-22 alpha, SM-MHC, VEGFA. Briefly, scaffolds were blocked using 5% BSA in 0.25% of Triton X for 1hr in room temperature followed by incubation with primary Ab for overnight at 4°C. After three washes in PBST (tween20 0.05%) secondary antibodies tagged with either Alexa Fluor® 568 or 488 were used. Dapi was used to stain the nuclei. The sections were washed three times with PBST for 5min each before fluorescence imaging.

Immunohistochemistry: Harvested tissue samples were paraffin embedded and cut into cross-sections of 5μm thickness. Immunostaining was performed using markers: human HIF1 alpha , VEGF, calponin, Ki67 and HLA and mouse CD31 (More information on primary antibodies can be found in the supplementary information). Briefly, tissue sections from the paraffin embedded samples were deparaffinized and processed through a gradient from 100% ethanol to 50% and finally in water. Antigen retrieval was carried out at 100°C using sodium citrate buffer (10mM sodium citrate, 0.05% Tween-20, pH 6.0). The tissue sections were blocked using 5% BSA in 0.25% of Triton X for 1hr in room temperature followed by incubation with primary antibody for overnight at 4°C. After three washes in PBST (tween20 0.05%) secondary antibodies tagged with either Alexa Fluor® 568 or 488 were used for 1hr. Dapi was used to stain nuclei. The sections were washed three times for 5 minutes each before fluorescence imaging.

Table 1. Information related to fabrication of hydrated collagen scaffolds of various density (total volume 500μl).

Density	5mg/ml of Rat tail Type-I Collagen (μl)	10x MEM (μl)	1M NaOH (μl)	SmGM-2 (μl)	Cells (8000/μl)
**1.25mg/ml**	125	50	2.3	300	25
**2.5mg/ml**	250	50	3	175	25
**4mg/ml**	400	75	8.5	0	25

Table 2. Information related to primary antibodies.

Primary Antibody	Dilution	Catalog Number/ Manufacturer
Angiopoietin-1	1:2500 ELISA	MAB9231-100 (R&D)
MMP-2	1:2500 ELISA<br>1:200 IHC	AF902 (R&D)
IL-8	1:2500 ELISA	MAB208 (R&D)
SDF-1α	1:2500 ELISA	MAB350 (R&D)
CD31	1:200 IHC	AF3628 (R&D)
PDGFAA	1:2500 ELISA	500-P46-100 (PeproTech)
KGF	1:2500 ELISA	500-P19-100 (PeproTech)
bFGF	1:2500 ELISA	500M38 (PeproTech)
TGFβ	1:2500 ELISA	SC-52893 (SantaCruz Biotech)
HIF-1α	1:2500 ELISA<br>1:200 IHC	SC-13515 (SantaCruz Biotech)
ICAM-1	1:200 IF and IHC	SC-8439 (SantaCruz Biotech)
Caspase-3	1:200 IF	SC-271028 (SantaCruz Biotech)
α -SMA	1:200 IF<br>1μg/ml FACS	SC-32251 (SantaCruz Biotech)
IL-10	1:2500 ELISA; 1:200 IHC	AB-34843 (Abcam)
VEGF	1:2500 ELISA; 1:200 IHC	AB-119 (Abcam)
SM-22α	1:300 IF and IHC<br>1μg/ml FACS	AB-10135 (Abcam)
SM-MHC	1:300 IF and IHC<br>1μg/ml FACS	AB-53219 (Abcam)
Ki67	1:300 IF<br>I:200 IHC	AB-92742 (Abcam)
HLA	1:200 IHC	AB-52922 (Abcam)
Caveolin-1	1:400 IF	610057 (BD Bioscience)
Calponin	1:200 IF and IHC<br>1μg/ml FACS	C-2687 (Sigma)

Table 3. Information related to secondary antibodies.

Secondary Antibody	Dilution	Catalog Number/ Manufacturer
Anti-Mouse-HRP	1:2500 ELISA	AB6789 (Abcam)
Anti-Rabbit-HRP	1:2500 ELISA	A0545 (Sigma)
Anti-Goat-HRP	1:2500 ELISA	HAF109 (R&D)
Anti-Mouse-Alexafluor488	1:400 IF and IHC	A11029 (ThermoFisher)
Anti-Mouse-Alexafluor568	1:400 IF and IHC	A11031 (ThermoFisher)
Anti-Goat-Alexafluor555	1:400 IF and IHC	A21432 (ThermoFisher)
Anti-Goat-Alexafluor488	1:400 IF and IHC	A27012 (ThermoFisher)
Anti-Rabbit-Alexafluor555	1:400 IF and IHC	A31572 (ThermoFisher)
Anti-Rabbit-Alexafluor488	1:400 IF and IHC	A11070 (ThermoFisher)

Table 4. Culture medium information for primary cells.

Cell Type	Medium	Catalog Information
Human Umbilical Endothelial Cell (HUVEC)	Endothelial Cell Growth Medium-2	PromoCell (C-22011)
Human Skin Fibroblasts	DMEM High Glucose and 10% FBS	Gibco (11965084; 26140079)
Human Epidermal Keratinocytes	EpiLife™	Gibco (MEPI500CA, S0125)

Figures:

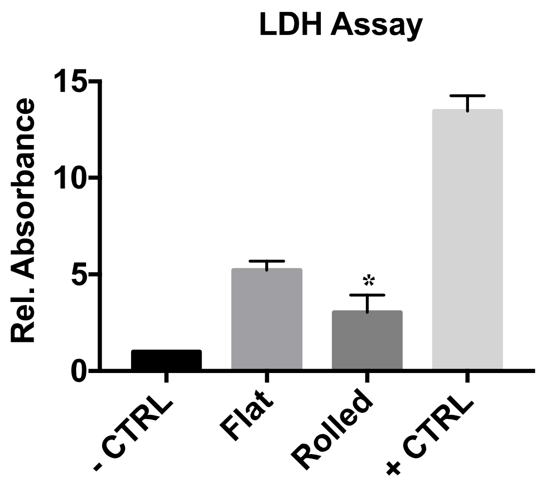

Supplementary Figure S1: Cytotoxicity characterization of hypoxic collagen scaffold on iPSC-VSMC: LDH assay was performed on lysates obtained from different scaffold culture conditions. The graph showed relative absorbance obtained from rolled hypoxic scaffold in comparison to different group of specimens including acellular scaffold as negative control, flat scaffold and positive LDH control (n=4, Rolled vs Flat $p< 0.009$; Rolled vs Positive control $p< 0.0001$). The statistical significance was obtained using one-way ANOVA. * denotes statistical significance.

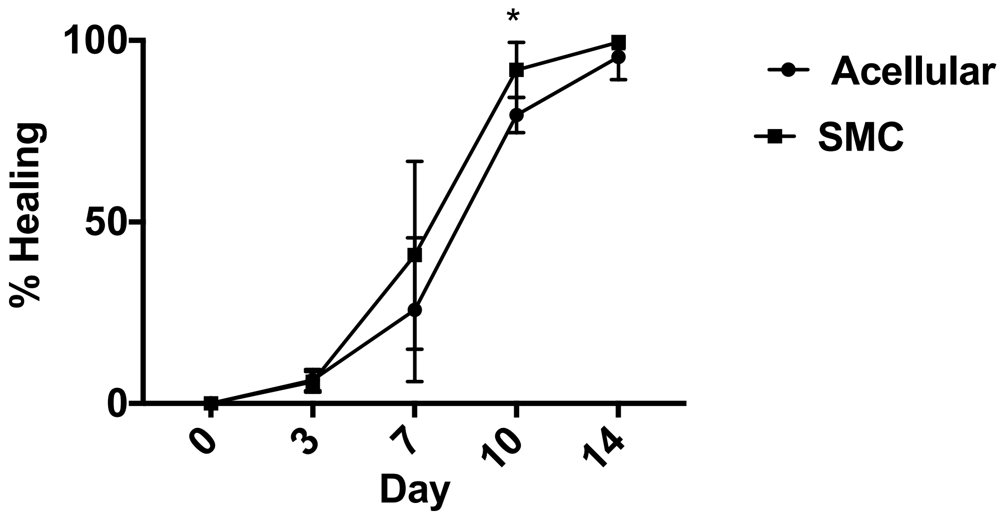

Supplementary Figure S2: Rolled DC scaffold promotes wound healing *in vivo* nude mice models: The hiPSC-VSMCs cultured in rolled scaffolds for 72 h were implanted in splinted back wound in nude mice and were observed on day 0, 3, 7, 10 and 14 day. Acellular scaffolds were used as control groups. The graph represents percent wound closure of the treatment groups acellular and hiPSC-VSMC-based scaffolds (n=6, hiPSC-VSMC vs Acellular p=0.007). * denotes statistical significance.
